# Type 2 diabetes impairs odour detection, olfactory memory and olfactory neuroplasticity; effects partly reversed by the DPP-4 inhibitor Linagliptin

**DOI:** 10.1186/s40478-018-0517-1

**Published:** 2018-02-23

**Authors:** Grazyna Lietzau, William Davidsson, Claes-Göran Östenson, Fausto Chiazza, David Nathanson, Hiranya Pintana, Josefin Skogsberg, Thomas Klein, Thomas Nyström, Vladimer Darsalia, Cesare Patrone

**Affiliations:** 1 Department of Clinical Science and Education, Södersjukhuset, Internal Medicine, Karolinska Institutet, Stockholm, Sweden; 2 Department of Molecular Medicine and Surgery, Karolinska Institutet, Stockholm, Sweden; 3 Department of Anatomy and Neurobiology, Medical University of Gdansk, Gdansk, Poland; 4 Boehringer Ingelheim AB, Stockholm, Sweden; 5 Boehringer Ingelheim Pharma GmbH & Co. KG, Biberach, Germany

**Keywords:** Diabetes, DPP-4 inhibitors, Goto-Kakizaki rats, Olfaction, Neuroplasticity, Piriform cortex

## Abstract

Recent data suggest that olfactory deficits could represent an early marker and a pathogenic mechanism at the basis of cognitive decline in type 2 diabetes (T2D). However, research is needed to further characterize olfactory deficits in diabetes, their relation to cognitive decline and underlying mechanisms.

The aim of this study was to determine whether T2D impairs odour detection, olfactory memory as well as neuroplasticity in two major brain areas responsible for olfaction and odour coding: the main olfactory bulb (MOB) and the piriform cortex (PC), respectively. Dipeptidyl peptidase-4 inhibitors (DPP-4i) are clinically used T2D drugs exerting also beneficial effects in the brain. Therefore, we aimed to determine whether DPP-4i could reverse the potentially detrimental effects of T2D on the olfactory system.

Non-diabetic Wistar and T2D Goto-Kakizaki rats, untreated or treated for 16 weeks with the DPP-4i linagliptin, were employed. Odour detection and olfactory memory were assessed by using the block, the habituation-dishabituation and the buried pellet tests. We assessed neuroplasticity in the MOB by quantifying adult neurogenesis and GABAergic inhibitory interneurons positive for calbindin, parvalbumin and carletinin. In the PC, neuroplasticity was assessed by quantifying the same populations of interneurons and a newly identified form of olfactory neuroplasticity mediated by post-mitotic doublecortin (DCX) + immature neurons.

We show that T2D dramatically reduced odour detection and olfactory memory. Moreover, T2D decreased neurogenesis in the MOB, impaired the differentiation of DCX+ immature neurons in the PC and altered GABAergic interneurons protein expression in both olfactory areas. DPP-4i did not improve odour detection and olfactory memory. However, it normalized T2D-induced effects on neuroplasticity.

The results provide new knowledge on the detrimental effects of T2D on the olfactory system. This knowledge could constitute essentials for understanding the interplay between T2D and cognitive decline and for designing effective preventive therapies.

**Electronic supplementary material:**

The online version of this article (10.1186/s40478-018-0517-1) contains supplementary material, which is available to authorized users.

## Introduction

Cognitive decline, dementia and Alzheimer’s disease (AD) are often preceded by olfactory deficits [reviewed in [18, 20]]. Interestingly, some studies show that type 2 diabetic (T2D) patients present olfactory impairments such as elevated odour detection threshold [39], reduced odour-identification ability [26, 51, 68], and increased risk of anosmia [9]. Since there is also a strong association between T2D and different forms of cognitive decline and dementia, including AD [6, 14, 40, 42, 90], olfactory dysfunction in T2D could represent an early indicator and perhaps even one of the pathogenic mechanisms at the base of future cognitive impairment. A few recent studies support this hypothesis [82, 91]. However, other studies could not detect olfactory deficits in diabetes [2, 9, 71] and these discrepancies call for further investigation. Moreover, the potential impairment of cognitive functions related to olfaction (e.g *olfactory memory*) needs to be studied in T2D, since the disruption of these functions in non-diabetics has been associated with aging and cognitive decline [12, 13, 50, 75, 85, 92].

The mechanisms at the basis of impaired olfaction in T2D have been poorly investigated, although some interesting studies have been performed in animal models of obesity/pre-diabetes. Livingston et al. have shown that obese, insulin-resistant rats have a decreased level of tyrosine-phosphorylated proteins in the main olfactory bulb (MOB) and piriform cortex (PC) [57] which are the two brain areas responsible for olfaction [67] and odour coding [5, 25], respectively. Furthermore, insulin binding in the MOB of these rats is decreased [4].

The brain’s ability to reorganize neural circuits in order to adapt to the environmental changes is called *neuroplasticity*. Olfaction requires *neuroplasticity* for both detecting and coding new odours and GABAergic inhibitory interneurons play an important role in this context [36]. Interestingly, the vulnerability of GABAergic inhibitory interneurons in the olfactory system has been associated with AD [79]. The potential effects of T2D on the interneuron-mediated *neuroplasticity* in the olfactory system have been investigated only in one study showing that calbindin (CB) + interneurons are affected by T2D [56].

Olfactory *neuroplasticity* is also regulated by adult neurogenesis in the MOB. This process occurs during the adult life and starts in the subventricular zone (SVZ) bordering the lateral ventricle. In the SVZ, neural stem cells (NSCs) produce undifferentiated and proliferative doublecortin (DCX) + neuroblasts that migrate towards the MOB where they differentiate mainly into interneurons playing an important role in the *neuroplasticity* of the MOB [31, 81]. While the detrimental effects of T2D on the NSCs in the SVZ have been recently shown [3, 49, 59, 60], it remains to be determined whether neurogenesis in the MOB is affected by T2D.

Another form of *neuroplasticity* in the olfactory system is represented by DCX+ immature neurons in the PC*.* In contrast to DCX+ cells in the MOB (see above), these cells are post-mitotic, non-proliferative immature neurons of embryonic origin. The pool of these cells decreases during aging due to continuous differentiation into mature neurons following new olfactory learning demands [41, 66]. Whether T2D affects these cells is unknown.

Recent studies suggest that olfactory deficits, in addition to their potential role as biomarkers, could also play an important role in the pathogenesis of AD [18, 21]. If so, the normalization of olfactory deficits in T2D could have a therapeutic preventive role against cognitive decline. Dipeptidyl peptidase-4 inhibitors (DPP-4i) are a growing class of clinically used T2D drugs [19, 83] that have also shown beneficial effects in the CNS of animal models of AD [15, 22, 45–47, 77] and in T2D patients with AD [37], even independently from glycemic regulation [27, 70]. Whether some of these beneficial effects occur via the normalization of impaired olfaction is unknown.

In this study, we addressed some of these issues in a lean and spontaneous model of T2D: the Goto Kakizaki (GK) rat [69]. Specifically, we investigated whether T2D impairs *odour detection* and *olfactory memory*. To determine whether T2D impairs the *neuroplasticity* in the olfactory system, we investigated whether GABAergic inhibitory interneurons and adult neurogenesis in the MOB as well as GABAergic interneurons and the immature DCX+ neurons in the PC are affected by T2D. Finally, we determined whether a chronic treatment with DPP-4i could reverse the identified factors affected by T2D.

## Materials and methods

### Animals and the T2D model

Rats were housed in 12-h light/dark cycle with free access to food and water. All experiments were conducted in accordance with the Guide for the Care and Use of Laboratory Animals published by U.S. National Institute of Health and approved by the regional ethics committee for animal experimentation (ethical permits granted by Stockholm’s Djurförsöksetiska Nämnd: S7–13 and N43/16).

As experimental model of T2D, the GK rat was used. This non-obese strain originates from selective breeding of Wistar rats and becomes spontaneously hyperglycemic during early adulthood [69]. The GK rats develop deficiency in insulin secretion and peripheral insulin/leptin resistance [73].

### Experimental design

**Fig. 1:**
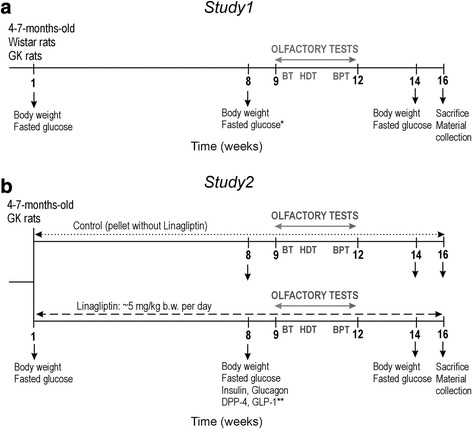
Experimental timeline of studies. Abbreviations: BT – block test, BPT – buried pellet test, DPP-4 – dipeptidyl peptidase 4, GK rat – Goto-Kakizaki rat, GLP-1 – glucagon-like peptide-1, HDT – habituation-dishabituation test. *Data presented in Additional file 1: Figure S1, **data presented in Additional file 1: Figure S3

In total, sixty five 4–7-months-old male GK rats and age-matched, non-diabetic Wistar rats were used in 2 studies:

*Study 1*: To determine the effect of T2D on *odour detection*, *olfactory memory* and *neuroplasticity*-related markers, we used T2D GK rats (*n* = 9) and Wistar rats (*n* = 11) as non-diabetic controls. The experimental design is summarized in Fig. 1a.

*Study 2*: To determine the potential effect of chronic DPP4i on the olfactory system, T2D GK rats (*n* = 23) were treated with linagliptin (mixed in the standard rodent chow at 83 mg/kg, estimated daily intake at ≈5–8 mg/kg/bw) for 16 weeks. Control GK rats (*n* = 22) received the chow of the same composition, but without the drug. *Odour detection*, *olfactory memory* and *neuroplasticity*-related markers were determined as in Study 1. The rationale for the dose of 5–8 mg/kg/bw and the duration of the treatment were to strongly and chronically inhibit DPP-4 activity. We employed middle-aged rats rather than old rats in the view to test the preventive potential of Linagliptin to decrease olfactory impairment. The experimental design is summarized in Fig. 1b.

### Body weight and glycemic level

Blood glucose levels (after 6 h of fasting; LifeScan glucometer, US) and body weight were measured in all animals. As previously published by us and others [33, 56, 73], results showed lower body weight (363.4 ± 9.3 vs. 503.4 ± 13.1, *p* < 0.0001) as well as increased hyperglycemia (11.8 ± 0.6 vs. 4.7 ± 0.2, *p* < 0.0001) in GK rats compared to Wistar controls (Additional file 1: Figure S1 A and B, respectively).

### Dipeptidyl peptidase 4 (DPP-4) activity and glucagon-like peptide-1 (GLP-1) levels

In order to verify the bioactivity of linagliptin, plasma DPP-4 activity and total active GLP-1 levels were determined by enzyme immunoassay (EIA) and by the GLP-1 assay kit (Meso Scale discovery, Gaithersburg, MD, USA).

### Olfactory assays

To assess the odour detection ability and the olfactory memory, the block test, the habituation-dishabituation test, and the buried pellet test were used [52]. The detailed description of the olfactory testing procedures and analyzed parameters are provided in the Additional file 1.

### Immunohistochemistry

For immunohistochemistry (IHC): the rats were deeply anesthetized with sodium pentobarbital and transcardially perfused with saline followed by 4% paraformaldehyde (PFA). Brains were post-fixed overnight in 4% PFA and placed in 20% sucrose solution until they sank. Brains were cut into 40 μm-thick coronal sections (apart from the olfactory bulb, which was cut in sagittal plane) using Leica SM2010 R sliding microtome (Leica, Germany).

The following primary antibodies were used: rabbit polyclonal against parvalbumin (PV) (1:1500, Abcam, UK), rabbit polyclonal against calbindin (CB) (1:400, Abcam, UK), rabbit monoclonal against calretinin (CR) (1:300, Vector Laboratories, US), goat polyclonal against doublecortin (DCX) (1:300, SantaCruz Biotechnology, US), mouse monoclonal against neuronal nuclei marker (NeuN) (1:300, Merck Millipore, US). Antigen retrieval was performed in 1 mM EDTA, pH 8.0 (for PV, CB and CR) in 70 °C for 35 min. After cooling, free-floating sections were incubated with primary antibodies overnight at 4°C in PBS containing 5% of natural horse serum (NHS) and 0.25% Triton-X100. Then, primary antibodies were detected by either fluorescent dye-conjugated [Alexa Fluor488 (DCX), Alexa Fluor594 (NeuN) (both 1:200, Life Technologies, US)] or biotin-conjugated secondary antibodies (1:200, Vector Laboratories, Sweden). The sections were incubated with the secondary antibodies for 2 h at RT in PBS containing 5% NHS and 0.25% Triton-X100. For chromogenic visualization, avidin-biotin complex (ABC kit, Vector Laboratories, Sweden) and diaminobenzidine (DAB; Sigma-Aldrich, US) were used.

To evaluate the density of projecting neurons in the mitral cell layer of the MOB, Nissl staining was performed.

### Quantitative analyses of IHC

The PV-, CB-, CR-, DCX/NeuN- positive cells were quantified using a computerized stereology toolbox equipped with Visiopharm v. 4.2.1.0 software for digital image analysis (NewCast, Denmark), connected to Olympus BX51 epifluorescent/light microscope (Olympus, Japan). Positive cells were counted on three coronal sections *per* animal located in 0.24, − 1.80 and − 3.36 mm distance to Bregma (PC) and two representative sagittal sections *per* animal (MOB). Accessory olfactory bulb was excluded from all assessments. The cell density per 1 mm^2^ was determined. Due to low number of DCX+ cells *per* section in PC and their characteristic localization (mostly in II layer), total number of DCX+ and DCX/NeuN+ cells in PC was estimated on sections located between − 1.20 and − 3.48 mm in relation to Bregma. Measurements of the mean cell volume (in μm^3^) of CB+ cells were made, using nucleator technique [28], by Visiopharm software. To assess the extent of interneuronal connectivity of CB+ cells, neuronal arborization (number of neurites/branching) was quantified [38]. Neuronal arborization was evaluated as showed in Additional file 1: Figure S2. Due to lack of visible neurites in CB+ cells in the MOB and negative preliminary results regarding differences in the mean volume of these interneurons, we present only the results of the arborisation/volume measurements for CB+ cells in the PC.

### Western blot

The PC and the MOB were dissected and snap frozen for further analyses. The tissue was homogenized in RIPA lysis buffer containing protease inhibitory cocktail (Sigma-Aldrich) on ice for 30 min. Total protein concentration was determined by Lowry assay (Bio-Rad Laboratories, US). Samples containing 4 μg/μl total protein were mixed with LDS sample buffer (Novex, Life Technologies, US) and incubated in 95 °C for 5 min. After electrophoresis on Bolt 4–12% Bis-TrisPlus gel (ThermoFisher Scientific, US), proteins were transferred on nitrocellulose membrane (iBlot 2 Dry Blotting System, 25 V for 6 min, ThermoFisher, US). Immuno-detection was performed with antibodies against: 1) PV (1:500, polyclonal, rabbit, Abcam, US), 2) CB (1:100 polyclonal, rabbit antibody, Abcam), 3) CR (1:1000, monoclonal, rabbit, Abcam, US), and 4) β-actin (1:450, monoclonal, mouse, SantaCruz Biotechnology). Secondary antibodies used: bovine anti-rabbit (1:10,000; SantaCruz Biotechnology) and goat anti-mouse (1:5000; SantaCruz Biotechnology, US) were conjugated with horseradish peroxidase (HRP). Immuno-reactive bands were developed using ECL substrate for HRP (GE Healthcare, Sweden) and documented with a GelDoc chemiluminescent system (Bio-Rad Laboratories, US). Normalization against β-actin was performed.

### Statistics

To analyze the data from behavioral experiments, nonparametric tests (Mann-Whitney *U*-test, Wilcoxon Signed Rank test) were employed to account for heterogeneity of variance. Additionally, for analyses of the behavioral data one-way and two-way ANOVA followed by appropriate post-hoc tests were applied (see Figure legends for detailed information). Cell density and relative protein content were compared using unpaired two-tailed *t* test. Multiple *t*-tests were applied to compare neuronal arborization. The statistical analyses were performed using GraphPad Prism 7 (USA). All data are presented as means ±S.E.M. and differences between the groups were considered significant when *p*-values were less than 0.05 (*^,Δ^*p* < 0.05; **^,ΔΔ^*p* < 0.01, *** *p* < 0.001, **** *p* < 0.0001).

## Results

### Odour detection and olfactory memory in T2D rats

#### T2D rats have deficits in odour detection ability and olfactory memory

**Fig. 2:**
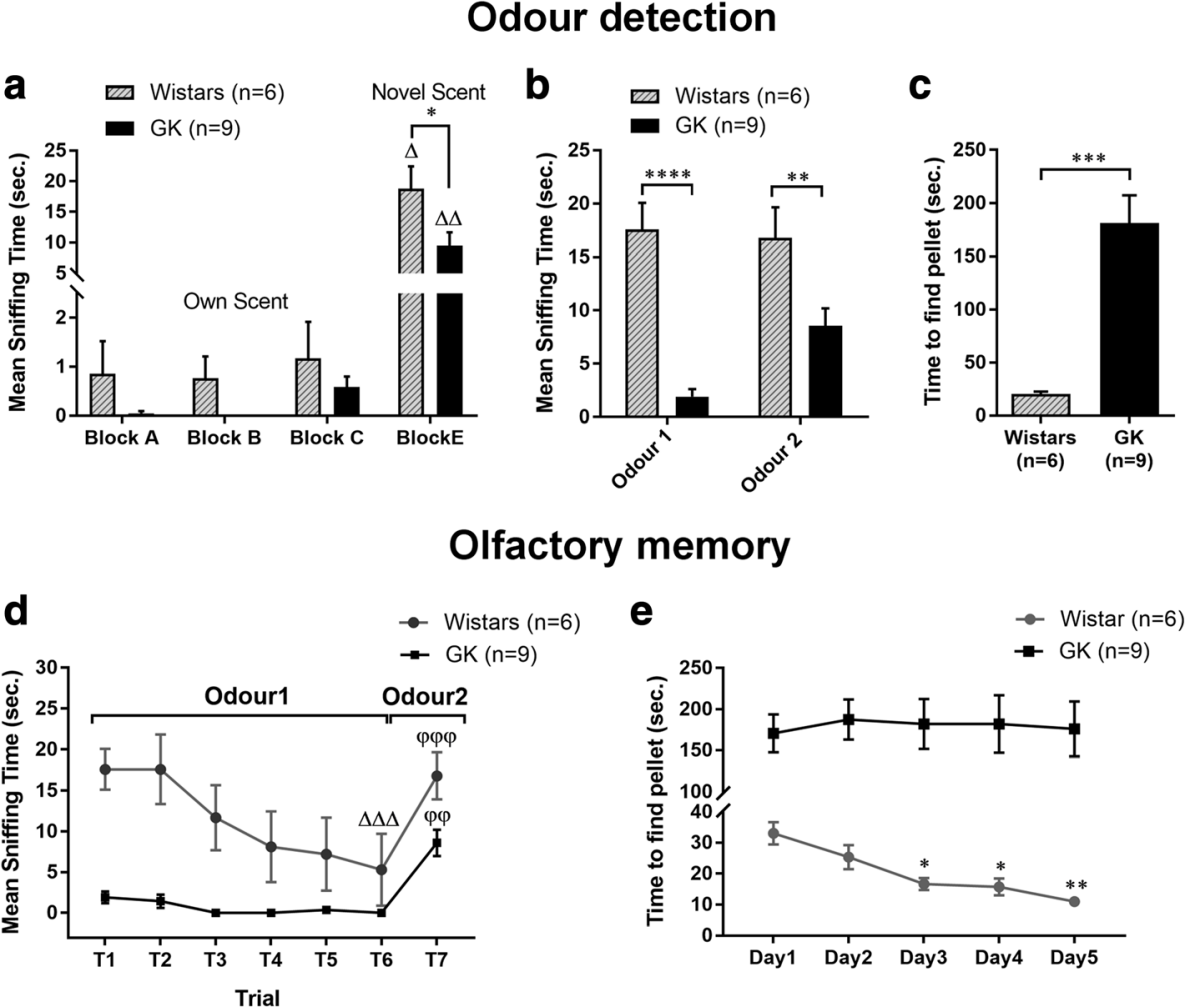
Diabetic rats show deficits in odour detection and olfactory memory. **a** Mean sniffing time of wooden blocks covered with a scent of the tested rat (blocks *A-C*) and unknown rat (block *E*) in the block test. *comparison of time spent sniffing block E between non-diabetic Wistar and T2D GK rats done by the Mann-Whitney *U*-test; ^∆^comparison of time spent sniffing block E with other blocks done by the Wilcoxon Signed Rank test. **b** Mean sniffing time of the scented cartridge covered with vanilla (odour 1) and lemon (odour 2) in the habituation-dishabituation test. Two-way ANOVA followed by Sidak’s multiple comparisons test. **c** Mean time to find pellet in the buried pellet test. Mann-Whitney *U*-test. **d** Mean sniffing time of the scented cartridge (vanilla/lemon) during 7 trials of the habituation-dishabituation test. Habituation was determined as significant difference in sniffing time between the first and last presentation of the same odour. ^∆^comparision of sniffing time of odour 1 between T1 and T6. Dishabituation was determined as significant difference between the last presentation of an odour and the first presentation of an alternative odour. ^φ^comparision of sniffing time between T6 (odour 1) and T7 (odour 2). Repeated measures One-way ANOVA followed by Bonferroni’s test. **e** Mean time to find pellet in buried pellet test performed during 5 consecutive days. Repeated measures One-way ANOVA with Greenhouse-Geisser correction followed by Bonferoni’s multiple comparisons test. The data are means ±S.E.M., *^, ∆^*p* < 0.05, **^, ∆∆, φφ^*p* < 0.01, ***^, Δ∆Δ, φφφ^*p* < 0.001, **** *p* < 0.0001

To assess the potential impairment of *odour detection* in T2D rats with confirmed hyperglycemia (see Materials and methods), we measured the mean sniffing time for various odours and the time to find a fragrant object. The results show that GK rats, compared to non-diabetic controls, spent less time sniffing new odours in the block test (8.5 ± 2.2 vs. 19.66 ± 5.1 s., *p* = 0.04, Fig. 2a) and the habituation-dishabituation test (odour 1 = vanilla: 1.9 ± 0.7 vs. 17.6 ± 2.5 s., *p* < 0.0001; odour 2 = lemon: 8.6 ± 1.6 vs. 16.8 ± 2.9 s., *p* = 0.01, Fig. 2b). GK rats also needed much more time to find the fragrant object in the buried pellet test (181.2 ± 26.1 vs. 20.3 ± 2.3 s., *p* = 0.0003, Fig. 2c).

To assess *olfactory memory*, we repeatedly measured the mean sniffing time for the same odour (the habituation-dishabituation test) and the time to find the fragrant object (the buried pellet test). The results show that during consecutive trials, GK rats did not improve neither sniffing time (ie. they did not habituate) nor time to find the fragrant object (Fig. 2d and e, respectively) in comparison to Wistar rats. However, both strains discriminate between odour 1 and 2. (Fig. 2d). No differences in time to approach the scented object (Additional file 1: Figure S3A and B) or in movement activity (Additional file 1: Figure S3 C and D) were detected between GK and Wistar rats in both olfactory tests.

Overall, these results indicate that T2D is associated with to a dramatic reduction in *odour detection* and *olfactory memory*.

### The effect of T2D on neuroplasticity in the MOB and PC

#### T2D affects CB+ interneurons in the MOB

**Fig. 3:**
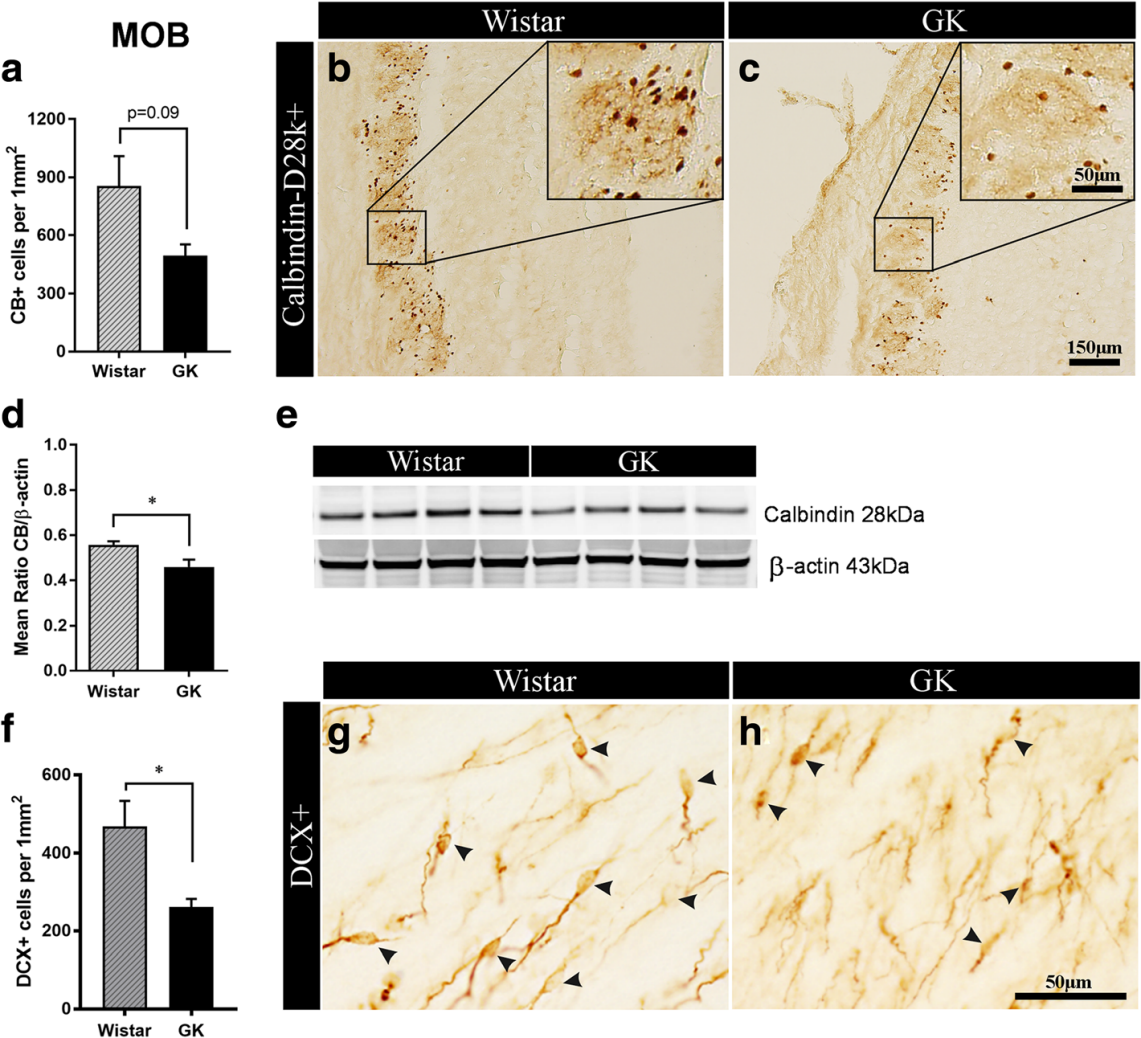
Diabetes affects calbindin+ interneurons and impairs adult neurogenesis in the olfactory bulb. **a** Density of CB+ interneurons. **b**-**c** representative microphotographs of CB+ staining in the MOB of non-diabetic Wistar versus T2D GK rats (*n* = 5–6). **d** Relative level of CB expression normalized against beta-actin and, **e** representative bands showing CB expression level in the MOB of non-diabetic vs. T2D rats (*n* = 4), (Western blot). **f** Density of immature DCX+ neurons and, **g**-**h** representative microphotographs of DCX+ staining in the MOB of Wistar vs. GK rats (*n* = 7) (black arrows indicate DCX+ cells). Two-tailed, unpaired *t*-test. The data are means ±S.E.M., * *p* < 0.05

We did not observe major structural changes in the MOB of GK and Wistar rats (e.g. by quantifying the mitral cells; data not shown). However, when we quantified CB+ interneurons in the MOB, the results show a trend, albeit not statistically significant, towards a decrease in the density of CB+ interneurons in diabetic compared with control rats (*p* = 0.09; Fig. 3a-c). Moreover, results show 18% decrease in CB expression in the MOB of GK rats versus Wistars by Western blot analysis (*p* = 0.04; Fig. 3d-e). Difference in neither the cell density nor expression of other interneuronal markers (i.e. PV and CR) between GK and Wistar rats was found (data not shown).

#### T2D reduces neurogenesis in the MOB

Adult neurogenesis in the MOB was assessed by counting DCX+ neuroblasts in this brain area. The results showed a dramatic reduction (over 50% decrease) in the density of DCX+ immature neurons in the MOB of GK compared with Wistar rats (261.7 ± 20.3 vs. 468.5 ± 65.9 cell/mm^2^, *p* = 0.02, Fig. 3f-h) indicating that MOB adult neurogenesis is severely impaired by T2D.

#### T2D impairs PV+ interneurons in the PC

**Fig. 4:**
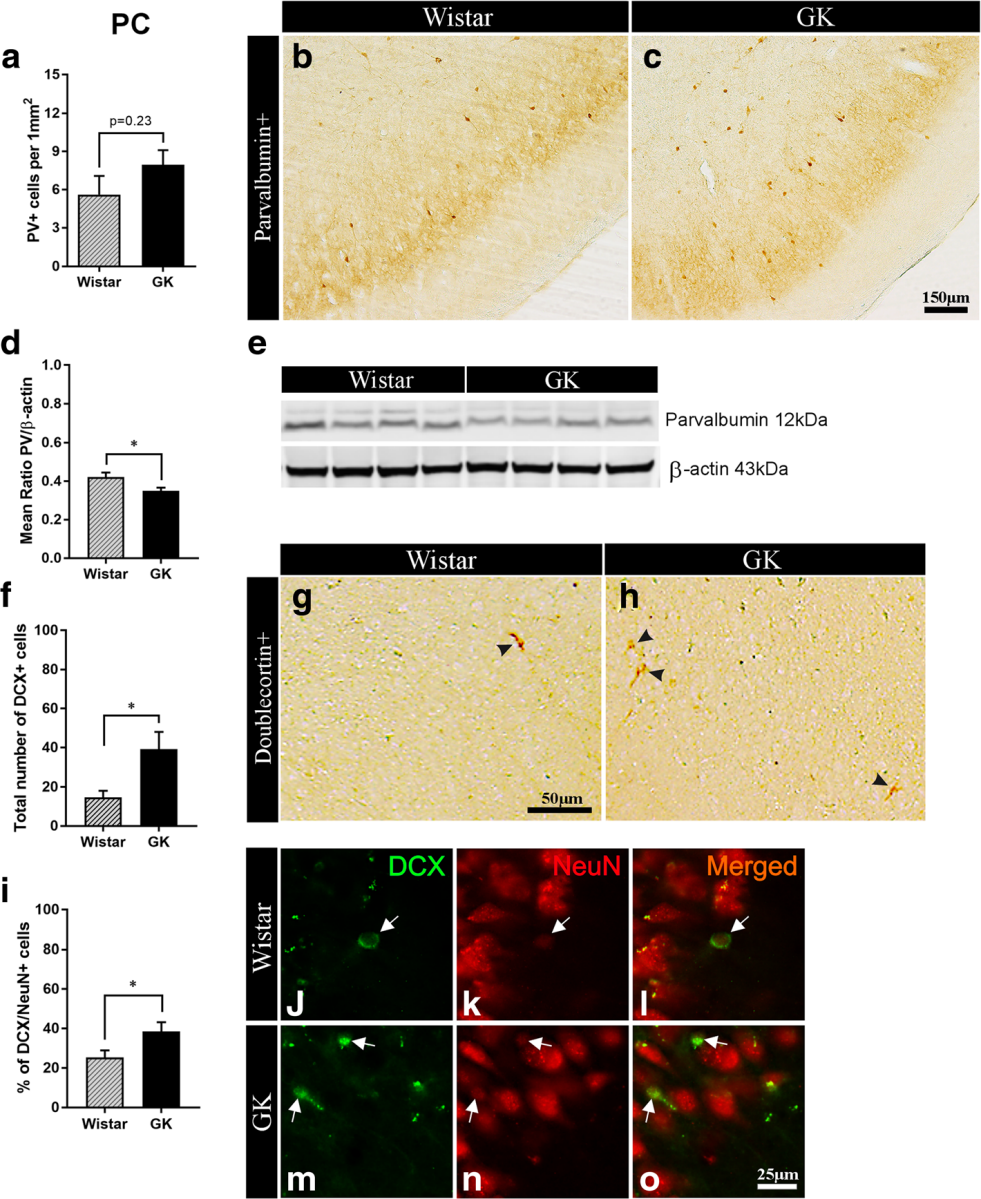
Diabetes decreases parvalbumin expression and impairs neuroblast differentiation in the piriform cortex. **a** Density of PV+ interneurons and, **b**-**c** representative microphotographs of PV+ staining in the PC of non-diabetic Wistar versus T2D GK rats (*n* = 7). **d** Relative level of PV normalized against beta-actin and, **e** representative bands showing PV expression level in the PC of non-diabetic vs. T2D rats (*n* = 4) (Western blot). **f** Total number and, **g**-**h** representative microphotographs of post-mitotic immature DCX+ neurons of embryonic origin in the PC of middle-aged Wistar vs. GK rats. **i** Percent of "differentiating" DCX/NeuN+ neurons and, **j**-**o** representative microphotographs of double-stained DCX/NeuN+ neurons (white arrows) in the PC of Wistar vs. GK rats (*n* = 7–9). Two-tailed, unpaired *t*-test. The data are means ±S.E.M., * *p* < 0.05

In our previous study, we showed vulnerability of GABAergic CB+ interneurons in the PC of GK rats during aging [56]. In the present study, we investigated the potential effect of T2D on PV+ and CR+ interneurons in the PC. We did not observe any significant difference in the density of PV+ interneurons between T2D and control rats (Fig. 4a-c). Results showed, however, 17% decrease in the expression of PV in the PC of diabetic compared with non-diabetic rats (*p* = 0.046; Fig. 4d, e), by Western blot analysis. No difference in either density of CR+ cells, or expression of this protein between GK and Wistars was recorded (data not shown). In summary, these results indicate that T2D affects PV+ interneurons in the PC.

#### T2D impairs the neuronal differentiation of DCX+ immature neurons in the PC

To assess the *neuroplasticity* related to adaptation to new odours, we quantified post-mitotic DCX+ immature neurons in the PC of 6 months-old T2D GK and non-diabetic rats. The results show that the total number of DCX+ cells was significantly higher (168% increase) in GK versus Wistar rats (39.2 ± 8.8 vs. 14.6 ± 3.4 cells; Fig. 4f-h). To determine whether the increase of DCX+ cells in GK rats corresponded to a decrease in neuronal differentiation, we double-stained DCX+ cells with the mature neuronal marker NeuN which is acquired by these cells during the differentiation process. Interestingly, the results show 52% increase in DCX/NeuN+ cells in the PC of the middle-aged T2D GK rats versus non-diabetic control rats (38.5 ± 4.7 vs. 25.3 ± 3.6 cells, *p* = 0.049; Fig. 4i-o). Overall, the results indicate an impairment of the differentiation rate of DCX+ cells (and thus of this form of *neuroplasticity*) in the PC of T2D rats.

### Odour detection and olfactory memory in GK rats treated with the DPP-4i linagliptin

Chronic linagliptin treatment led to a 55% reduction in peripheral DPP-4 activity (45.2 ± 2.3 vs. 100.0 ± 5.5% of control, *p* < 0.0001; Additional file 1: Figure S4A) and to increase active GLP-1 levels (0.66 ± 0.1 vs. 0.04 ± 0.002 pg/ml, *p* < 0.0001; Additional file 1: Figure S4B) versus the untreated controls. These results demonstrate the bioactivity of linagliptin. However, the chronic linagliptin treatment did not affect the glucagon and insulin concentrations, body weight, and glycemic levels (Additional file 1: Figure S4C-F). One possibility to explain these results is that linagliptin was less efficient in reducing DPP-4 activity (55% reduction in our study vs. more than 80% reduction in the previous studies [16, 17]).

#### Chronic DPP-4 inhibition does not improve odour detection and olfactory memory in T2D rats

**Fig. 5:**
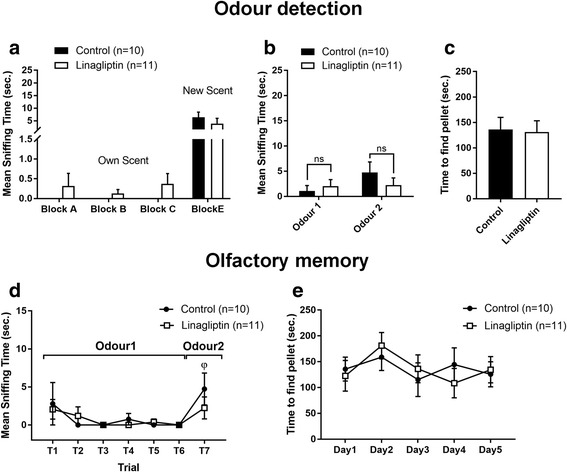
Chronic DPP-4 inhibition does not affect odour detection and olfactory memory in diabetic rats. **a** Mean sniffing time of wooden blocks covered with a scent of the tested rat (block *A-C*) and unknown rat (block *E*) in the block test. **b** Mean sniffing time of the scented cartridge covered with vanilla and lemon odour in the habituation dishabituation test. **c** Mean time to find pellet in the buried pellet test. **d** Mean sniffing time of the scented cartridge (vanilla/lemon) during 7 trials of the habituation dishabituation test. **e** Mean time to find pellet in the buried pellet test performed during 5 consecutive days. For comparisons between linagliptin-treated and untreated GK rats in this Study 2, the same statistical tests were performed as in Study 1 (see Fig. 2). The data are expressed as means ±S.E.M., ^φ^*p* < 0.05

Chronic treatment of GK rats with linagliptin for 16 weeks did not improve *odour detection* (Fig. 5a and b) and did not shorten the time to uncover the food (Fig. 5c). Similarly, the results showed no difference in the *olfactory memory* between linagliptin and control-treated GK rats (Fig. 5d and e). Interestingly, while untreated GK rats discriminated between the odours, linagliptin-treated rats did not. No significant difference in the approach time to the fragrant object as well as the movement activity between the linagliptin-treated and control GK rats was recorded (data not shown).

### The effect of chronic DPP-4 inhibition on neuroplasticity in the MOB and PC of T2D rat

#### Chronic DPP-4 inhibition normalizes CB+ interneurons in the MOB of T2D rats

**Fig. 6:**
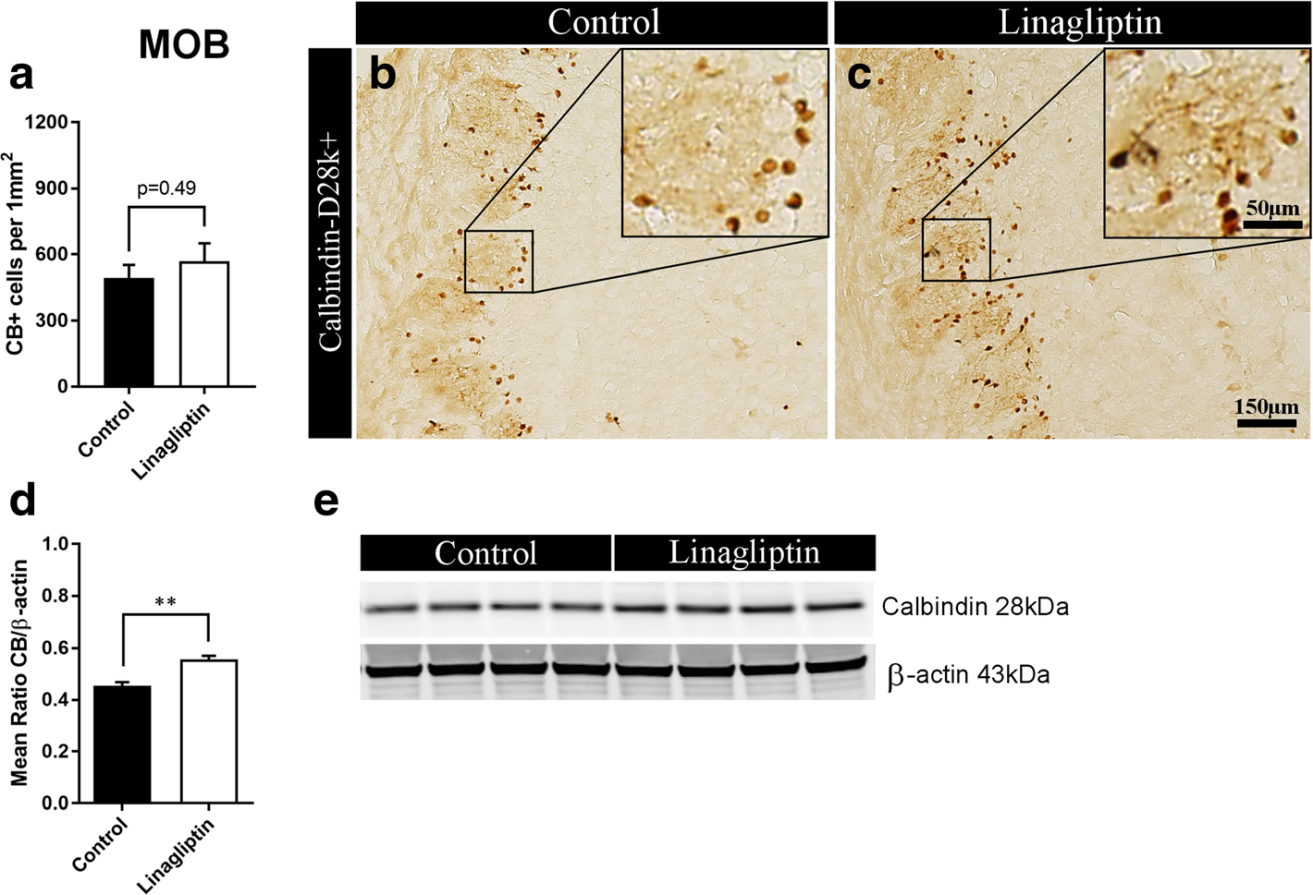
Chronic DPP-4 inhibition increases calbindin expression in the olfactory bulb of diabetic rats. **a** Density of CB+ interneurons and, **b**-**c** representative microphotographs of CB+ staining in the MOB of control vs. linagliptin-treated GK rats (*n* = 6). **d** Relative level of CB normalized against beta-actin and, **e** representative bands showing CB expression level in the MOB of control vs. linagliptin-treated GK rats (*n* = 4), (Western blot). Two-tailed, unpaired *t*-test. The data are means ±S.E.M., ** *p* < 0.01

In the MOB, no significant difference in the density of CB+ interneurons was observed in untreated versus linagliptin-treated GK rats (Fig. 6a-c). However, the results show 22% increase (*p* = 0.002; Fig. 6d and e) in CB expression in the MOB after chronic treatment with linagliptin compared to the control group. No significant differences in neither cell density nor expression of PV- and CR-positive interneurons were observed (data not shown). These results suggest that DPP-4i can normalize the T2D-induced down-regulation of CB in the MOB (see Fig. 3d).

#### Chronic DPP-4 inhibition has no effect on adult neurogenesis in the MOB of T2D rats

No difference in the density of DCX+ cells was observed in the MOB of linagliptin-treated versus control rats (data not shown) indicating no effect of this drug on adult neurogenesis in the MOB.

#### DPP-4 inhibition normalizes CB+ interneurons in the PC of T2D rats

**Fig. 7:**
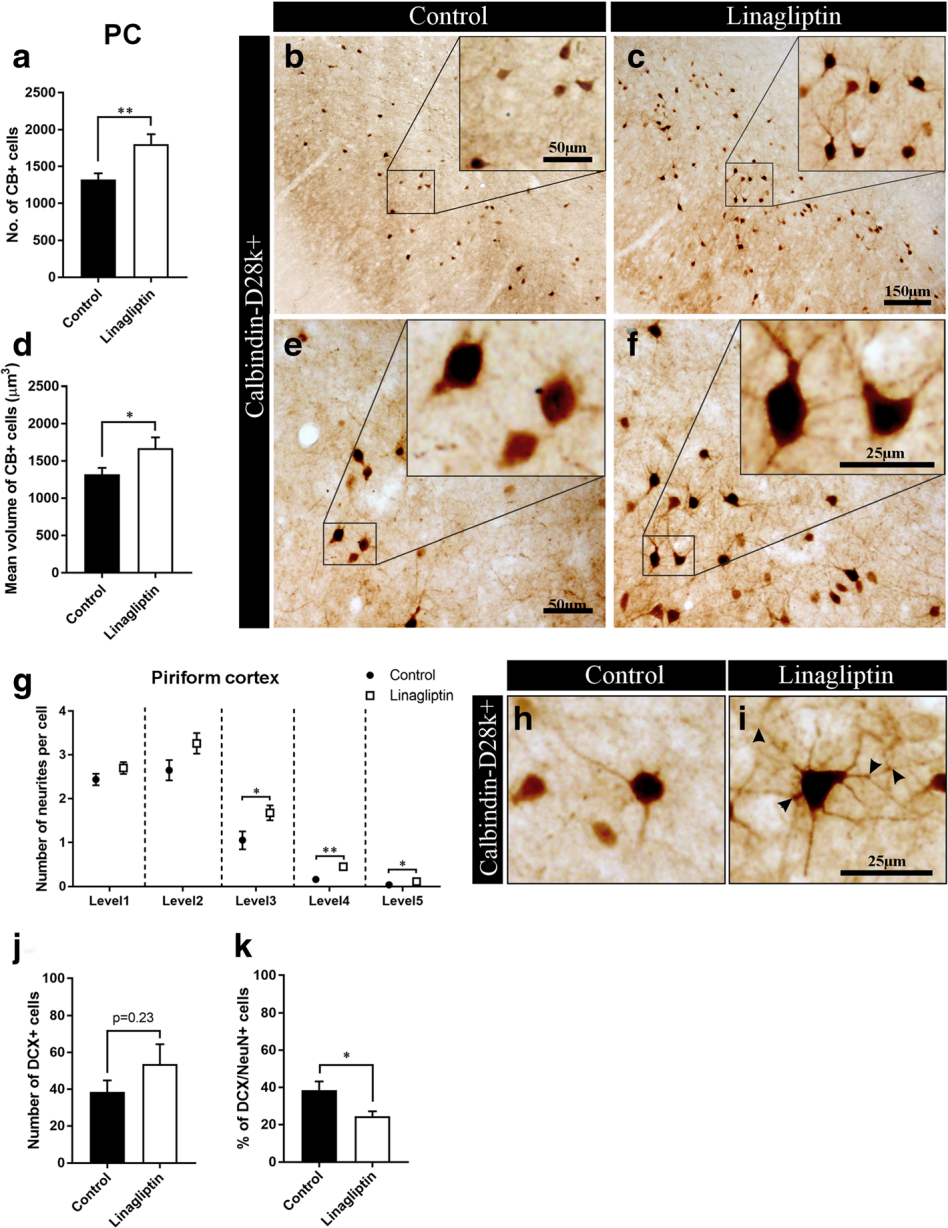
Chronic DPP-4 inhibition increases the number, the mean volume, and the arborization of calbindin+ interneurons and it promotes neuroblast differentiation in the piriform cortex of diabetic rats. **a** Density of CB+ interneurons and, **b**-**c** representative microphotographs of CB+ staining in the PC of control vs. linagliptin-treated GK rats (*n* = 9). **d** Mean volume and, **e**-**f** illustrating microphotographs of CB+ interneurons in the PC of control vs. linagliptin-treated GK rats. **g** Neuronal arborization and, **h**-**i** illustrating microphotographs of CB+ interneurons in the PC of the compared groups (*n* = 9). **j** Total number of DCX+ immature neurons and, **k** percent of double-stained DCX/NeuN+ "differentiating" neurons (*n* = 8). Two-tailed, unpaired *t*-test. The data are means ±S.E.M., * *p* < 0.05, ** *p* < 0.01

In the PC, the results showed over 40% increase in the number of CB+ interneurons after chronic linagliptin treatment compared to the control group (1849 ± 124 versus 1278 ± 86 cells, *p* = 0.002) (Fig. 7a-c) suggesting a significant role of DPP-4i in the regulation of *neuroplasticity* driven by these cells in the PC. The results showed no difference between control and linagliptin-treated group in neither the density of both PV- and CR+ interneurons nor the expression of these interneuronal markers (data not shown).

#### DPP-4 inhibition exerts neurotrophic effects on CB+ interneurons in the PC of T2D rats

Interestingly, the average soma volume of CB+ interneurons in the PC of the linagliptin-treated GK rats was 14% larger compared to the untreated rats (1567 ± 56 versus 1373 ± 49 μm^3^, *p* = 0.02; Fig. 7d-f). Moreover, the results show differences in the neuronal arborization of the CB+ interneurons in the PC after chronic treatment with linagliptin. Specifically, the number of neuronal branches per cell (see Material and Methods for the quantification procedures) was significantly increased in the PC of the linagliptin-treated animals compared to control group (level 3: 1.67 ± 0.17 vs. 1.05 ± 0.2, *p* = 0.03; level 4: 0.45 ± 0.06 vs. 0.16 ± 0.05, *p* = 0.002; level 5: 0.11 ± 0.02 vs. 0.04 ± 0.02, *p* = 0.02; Fig. 7g-i). Overall, these morphometric changes indicate a neurotrophic effect mediated by linagliptin on CB+ interneurons suggesting that DPP-4i regulate the *neuroplasticity* driven by CB+ interneurons in the PC.

#### DPP-4 inhibition promotes neuronal differentiation in the PC of T2D rats

Sixteen weeks of DPP-4 inhibition resulted in no significant difference in the number of DCX+ neurons in the PC of GK rats compared with control rats (Fig. 7j). However, when looking at the rate of their differentiation into mature neurons, linagliptin induced a strong 36%-decrease in DCX/NeuN double-stained neurons versus the control (24.5 ± 2.6 vs. 38.5 ± 4.7, *p* = 0.03; Fig. 7k). These results indicate that DPP-4 inhibition promotes the neuronal differentiation of these cells.

## Discussion

The primary aim of this study was to investigate the potential effects of T2D on *odour detection* and *olfactory memory*. Secondly, we determined whether important neuronal populations regulating the *neuroplasticity* in the two major brain areas involved in smelling and odour coding (the MOB and the PC) were affected by T2D. We show that T2D dramatically decreases *odour detection* and *olfactory memory*. These functional effects correlated with the decrease in CB expression and adult neurogenesis in the MOB. Moreover, T2D decreased PV expression and impaired the differentiation of DCX+ immature neurons in the PC. The third aim of the study was to determine whether a therapy mediated by DPP-4i could counteract the identified T2D effects on the olfactory system. While a chronic treatment with DPP-4i could not improve *odour detection* and *olfactory memory*, PV regulation in the PC and adult neurogenesis in the MOB, this pharmacological treatment could normalize CB+ interneurons in the MOB and PC. In addition, DPP-4i could exert neurotrophic effects on CB+ interneurons and promoted neuronal differentiation of immature DCX+ neurons in the PC.

### The effects of T2D on *odour detection*, *olfactory memory* and *olfactory neuroplasticity*

The relation between diabetes and olfactory deficits was shown for the first time already in the 60’s [39]. Since then a few other clinical studies have reported that diabetes could negatively impact olfactory functions [26, 51, 68]. However, a great limitation of these studies was the low number of patients. Moreover, discrepancies in the results of these studies, also in relation to differences between T1D and T2D [2, 9, 71], need to be clarified in order to undoubtedly prove the deleterious effects of T2D on the olfactory system. Importantly, the association between olfactory dysfunction in T2D with cognitive decline and dementia (including AD) has been suggested by a few clinical studies [82, 91]. However, the findings need to be further confirmed by employing additional and more reliable olfactory tests, since the ones used so far vary greatly in terms of sensitivity and reliability, as well as in the power to separate sensory and cognitive components of the olfactory functions. This is an important factor as suggested by a recent study by Markopoulou and colleagues who showed that individuals with olfactory dysfunction had poor inter-test consistency among the sets of odours identified incorrectly in successive replicate tests, even before severe olfactory dysfunction appeared [61]. Another important aspect, to prove the potential association of olfactory deficits in T2D with cognitive decline, is that olfactory parameters directly related to cognition, such as *olfactory memory* [12, 13, 24, 50, 75, 92] have, to the best of our knowledge, not been previously investigated.

In the attempt to address experimentally some of these questions, we employed a lean and spontaneous animal model of T2D, the GK rat. We used this model to rule out obesity-related factors. Furthermore, GK rats have recently been shown to present CNS complications and impaired mechanisms at the basis of cognition and memory [10, 33, 54–56, 62–65, 73]. Using three different functional tests, we show that T2D impairs *odour detection*. We also provide, for the first time, experimental evidence that *olfactory memory* is strongly impaired in T2D. Interestingly, recent works showed that hyperlipidemic and fructose-based diets disrupt odour-related learning [48, 74, 87]. These findings could be relevant for the understanding of the interplay between T2D and cognitive decline/dementia and they call for new clinical studies aimed at determining whether *olfactory memory* is impaired in T2D patients and, if so, whether this impairment correlates with the incidence of cognitive decline and dementia.

In the attempt to identify some of the pathophysiological mechanisms at the basis of impaired *odour detection* and *olfactory memory* in T2D, we investigated specific populations of neuronal cells involved in the regulation of the *neuroplasticity* of the MOB and the PC. We found decreased expression of CB (and a strong trend toward a decrease in the density of CB+ interneurons) in the MOB of GK rats. Interestingly, the vulnerability of CB+ interneurons in the AD brain has been known since the late 80’s [32, 34]. More recently, it has been suggested to represent a pathogenic mechanism at the basis of AD development [1, 43]. Importantly, decreased CB+ interneurons in the MOB of an AD mouse model has been recently shown [80]. To our knowledge, this is the first report showing that CB is decreased by T2D in the MOB. Although speculative, this data suggest that this decrease could represent an early step in the development of cognitive decline in T2D.

Adult neurogenesis in the MOB is an important cellular process at the basis of olfactory functions and olfaction-related *neuroplasticity* [31, 81]. NSC proliferation and survival in the SVZ (where NSCs are generated) are impaired by T2D [3, 49, 59, 60]. However, NSC proliferation and survival are only the initial steps of adult neurogenesis and thus cannot not fully depict the effect of T2D on this process. Immature neuroblasts derived from NSCs migrate from the SVZ to the MOB and it is here where they differentiate into neurons, i.e. the ultimate result of adult neurogenesis. Therefore, investigation of the potential effects of T2D on neurogenesis requires assessment of the neurogenesis process specifically within the MOB. Impairment of the MOB’s neurogenesis has been shown in one elegant study by Wakabayashi and colleagues with STZ-induced T1D [89]. However, MOB neurogenesis in T2D has not been previously investigated. Our results are in line with the results by Wakabayashi in T1D and they show a dramatic decrease in the number of DCX+ neuroblasts in the MOB of GK rats. Interestingly, deficits in adult neurogenesis in the MOB may mediate premature cognitive decline in AD [29]. This suggests that the impairment of this process in T2D could also represent an early pathogenic mechanism at the basis of cognitive decline in diabetics.

The next step in our study was to investigate potential changes induced by T2D in the PC, which is critical for odour coding [5]. The PC receives direct synaptic input from the MOB via the lateral olfactory tract and it is connected to higher brain centers such as the entorhinal cortex. Interestingly, the disruption of this region mediates early olfactory deficits in AD [53]. In the PC, the role of interneurons is fundamental for the synaptic inhibition after olfactory stimulation by a range of different odours [72, 93]. When investigating the potential effects of T2D on interneurons in the PC, we found that the expression of PV was decreased in GK rats. Importantly, PV+ interneurons play a crucial role in the regulation of cognition [11] and their dysfunction has been suggested to be a key mechanism in AD’s development [88]. Furthermore, PV+ interneurons are particularly vulnerable to AD in humans [8] and PV expression is lower in AD patients versus healthy controls [35]. Finally, a recent study by Saiz-Sanchez et al. showed that PV+ interneurons in the PC are up-regulated in the AD human brain [78]. Although electrophysiology experiments are needed to well characterize the functional role of PV+ interneurons in the PC, our data support the hypothesis that PV dysregulation in T2D could be linked to impaired *odour detection* and *olfactory memory*. This could represent an early pathogenic process at the basis of cognitive decline in T2D. These findings in the PC are supported by our recent work showing that impairment of PV+ interneurons in the hippocampus of T2D rats correlates with decreased gamma oscillations [55] which is an important mechanism at the basis of cognition.

The number of DCX+ immature neurons in the PC decreases along the course of life due to continuous differentiation into mature neurons. This pool of post-mitotic cells is generated prenatally [76] and, therefore, they are not derived from adult neurogenesis [66]. The knowledge at the basis of this process is largely unknown. However, it is believed that DCX+ cells represent a newly identified form of olfactory *neuroplasticity* in relation to olfactory learning and adaptation to new olfactory stimuli [7, 41, 84, 86]. Our results show that T2D dramatically reduces this process suggesting, for the first time, a potential negative effect of T2D on the adaptation to new odours. Whether the impairment of this form of *neuroplasticity* can be related to cognitive decline in T2D remains to be investigated.

### The effects of DPP-4i on *odour detection*, *olfactory memory* and olfactory *neuroplasticity* in T2D

In addition to be used clinically for the regulation of glycemia in T2D, DPP-4i mediate neurotrophic, neurogenic and neuroprotective effects in the brain, also independently of glycemia regulation [27, 70]. We investigated the potential efficacy of a chronic treatment with the DPP-4i linagliptin to counteract the identified functional and structural deficits induced by T2D on the olfactory system. We used linagliptin because the drug showed positive effects in animal models of neurodegenerative diseases [44] and cerebral stroke [16, 17].

Furthermore, glucose normalization was not obtained in GK rats (see Results), which allowed us to focus on potential glucose independent effects of the drug.

We show that linagliptin normalize some of the T2D-dysregulated *neuroplasticity* parameters (i.e. CB+ interneurons in the MOB and PC and immature DCX+ neurons in the PC). However, this treatment improved neither *odour detection* nor *olfactory memory* in T2D GK rats. Furthermore, linaglipin-treated rats showed impaired odour discrimination. One possibility to explain the lack of behavioral efficacy is that the employed olfactory tests in this study are not sensitive enough to detect pharmacologically-mediated improvements. New studies employing highly sensitive olfactometers could be useful in this respect as Thiebaud et al. have recently shown by studying the effects of a hyperlipidemic diet on olfaction in the mouse [87]. Another potential explanation is that the T2D-induced impairment in *odour detection* and *olfactory memory* is mainly driven by hyperglycemia (which is well known to have detrimental effects on the brain [58]), while *neuroplasticity* changes are glycemia-independent. In support of this hypothesis is the fact that Linagliptin was ineffective in the regulation of glycemia in the GK rat, as also recently shown [30]. If this is the case, the GK rat might not be the best model to study the effects of DPP-4i on *odour detection* nor *olfactory memory* and this is a weakness of our study. However, this aspect could also be a strength of this study allowing for the identification of glycemic-independent effects (on *neuroplasticity)* of T2D on the olfactory system. Additional studies investigating *odour detection* and *olfactory memory* in other T2D models, where DPP-4i efficiently regulate glycemia, will answer this question. Finally, another potential reason to explain the lack of effect of linagliptin on *odour detection* and *olfactory memory* is that low DPP-4 activity has been associated with inflammation of the nasal mucosa [23]. Although speculative, this effect could have masked a potential beneficial action of linagliptin on *odour detection* and/or *olfactory memory* and this is, in our sense, the most likely interpretation.

Despite the need to answer these questions by new studies, our results indicate that a chronic therapy mediated by DPP-4i can normalize the effect of T2D on CB+ interneurons in both the MOB and the PC by also inducing morphological and neurotrophic changes. This effect is potentially very interesting in relation to the established importance of these interneurons in AD progression [1, 32, 34, 43, 80]. Therefore, our results may suggest the preventive potential of DPP-4i to counteract these detrimental processes in the CNS. We also provided evidence that DPP-4i strongly (50%) decreases the number of DCX/NeuN double positive cells in the PC of GK rats. This indicates enhanced differentiation of immature DCX+ neurons into mature neurons and likely an improved capacity for adaptation to new smells. These results provide the first experimental evidence for the effect of DPP-4i on this newly identified form of *neuroplasticity* in the PC. Whether the improvement of this form of *neuroplasticity* can be associated with improved cognition in T2D remains to be investigated.

## Conclusions

In conclusion, the results of our study prove the detrimental effects of diabetes on *odour detection* in a lean model of T2D. Importantly, this study gains new knowledge about the detrimental impact of T2D on *olfactory memory* providing a strong rationale for testing this parameter in future clinical studies, e.g. as an early predictor for cognitive decline in T2D. We also show, for the first time, how T2D affects *neuroplasticity* in both the MOB and the PC by identifying the impairment of specific cellular mechanisms. These mechanisms could turn out to be key for understanding not only how T2D impairs olfaction but also the interplay between impaired olfaction in T2D and cognitive decline and dementia. While we could not demonstrate the efficacy of DPP-4i to improve *odour detection* and *olfactory memory*, our results show that linagliptin could improve cellular parameters related to the *neuroplasticity* in the olfactory system that were impaired by T2D. This provides new knowledge on the mechanisms at the basis of the beneficial effects of DPP-4 inhibitors in the T2D brain that could be exploited to design preventive therapies to treat cognitive decline in diabetes.

## Additional file

Additional file 1: Supplementary material. (DOCX 401 kb)
