# Supplementary material for: Type 2 diabetes impairs odour detection, olfactory memory and olfactory neuroplasticity; effects partly reversed by the DPP-4 inhibitor Linagliptin
Source: Acta Neuropathol Commun. 2018 Feb 23;6:14. doi: 10.1186/s40478-018-0517-1 (PMC5824492; doi:10.1186/s40478-018-0517-1)
Supplement: Supplementary file 1 — Supplementary material. (DOCX 401 kb) [file 40478_2018_517_MOESM1_ESM.docx]

**Type 2 diabetes impairs odour detection, olfactory memory and olfactory neuroplasticity; effects partly reversed by DPP-4 inhibition; *Acta Neuropathologica Communications***

## Grazyna Lietzau^1^, William Davidsson, Claes-Göran Östenson, David Nathanson, Hiranya Pintana, Josefin Skogsberg, Thomas Klein, Thomas Nyström, Vladimer Darsalia^1^, Cesare Patrone^1^

**Corresponding authors**: Grazyna Lietzau (PhD): [grazyna.lietzau@ki.se](mailto:vladimer.darsalia@ki.se); Vladimer Darsalia (PhD): [vladimer.darsalia@ki.se](mailto:vladimer.darsalia@ki.se); Cesare Patrone (Ph.D.): [cesare.patrone@ki.se](mailto:cesare.patrone@ki.se)

^1^Department of Clinical Science and Education, Södersjukhuset, Internal Medicine, Karolinska Institutet, Stockholm, Sweden

**Olfactory assays**

***Block test (BT)***

Preparations: rats were housed separately in clean cages (having free access to food and water) with 5 wooden blocks A-E for 24 hrs. During testing day, animals were habituated in their home cages without feeder bin and blocks for 1 hr. Blocks along with a handful of bedding were placed in a clean plastic bag labelled with rat's identification.

Testing procedure: Overall, seven trials were performed. During trials 1-6, blocks A-D from the animal's own cage were placed in the middle of the cage and rat's behavior recorded with a camera for 40 sec. After that time, recording was stopped, blocks removed from the cage and placed back into the plastic bag. The interval between trials lasted approx. 5 minutes. On the 7th trial, blocks A-C (from rat's own cage) + block E (from another rat's cage) were used. Then, animals were placed in a new clean cage with their original cagemates.

Analyzed parameters: 1) time spent by rat sniffing each block (sniffing defined as nasal contact with the block), 2) time to approach block E during trial7, and 3) total movement activity (time the rat was walking over the cage).

***Habituation-dishabituation test (HDT)***

Preparations: rats were housed separately in clean cages (having free access to food and water) with unscented cartridge (a small plastic container with openings) for 24 hrs. During testing day, animals were habituated in the test room, in their home cages without feeder bin and cartridge for 1 hr. Scented tissue cartridges were prepared in a separate room. To adjust concentration of each extract (Oetker, Sweden), we performed sensitivity test with rats not used in the study (dilutions 1:1, 1:10, 1:100, 1:1000). In the study was used 1:100 dilution, and 3µl of vanilla extract and 5µl of lemon extract were added on small cotton ball, inserted to clean cartridge, and placed in labelled, sealed plastic bag.

Testing procedure: Overall, seven trials were performed. During trials1-6, the cartridge with odour1 was placed in rat's cage and rat's behavior recorded with a camera for 40 sec. The interval between trials lasted approx. 5 minutes. On the 7th trial, new scent (odour2) was introduced. After the last trial, animals were placed in a new clean cage with their original cagemates.

Analyzed parameters: 1) time spent by rat sniffing the cartridge, 2) time to approach the cartridge during trial1 and 7, and 3) total movement activity.

***Buried pellet test (BPT)***

Preparations: Three days before testing, rats' weight was recorded and then food restricted to 90% of their body weight. Prior to testing, rats were introduced with a new pellet (piece of a protein bar). During all testing days, animals were habituated in the test room, in their home cages without feeder bin. For each tested animal, a clean cage was filled with clean bedding (~3cm) evenly distributed throughout the cage and the pellet buried ~0.5cm below it.

Testing procedure: The rat was placed in the center of the test cage and timer and recording camera set for 5 min. After that time, the rat was returned to its cage with the original cage mates. For each rat, the test was performed twice each day. Total number of testing days was 5. During each trial, the pellet was buried in a different spot in the cage.

Analyzed parameters: 1) time for the rat to uncover and start eating the pellet. If rat did not find the pellet within 5 min., the trial was ended and a score 300 sec. noted.

**
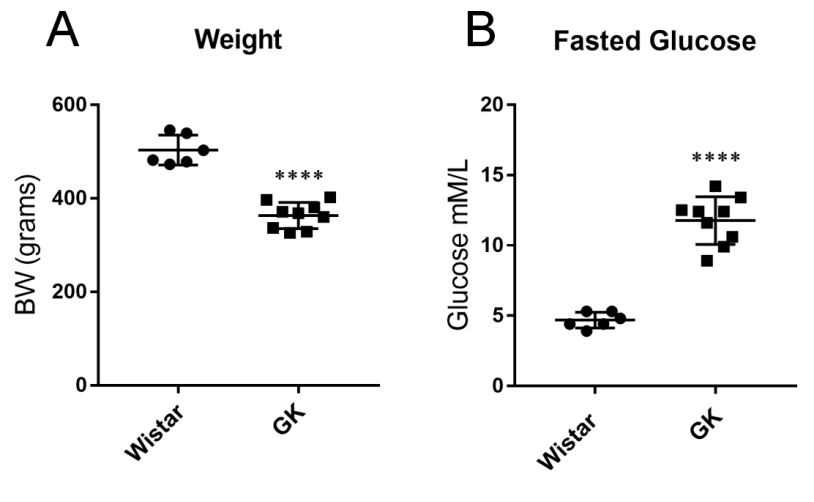
**

**Fig. S1 Metabolic parameters of non-diabetic vs. diabetic rats (Study1).** Body weight (**A**), and glucose concentration after 6h of fasting (**B**), in Wistar (n=6) and GK (n=9) rats. Two-tailed, unpaired *t*-test. The data are means ±S.E.M., **** p <0.0001

**
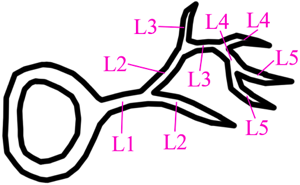
**

**Fig. S2 Schematic illustration of a neuron with neurite arborization up to level 5**

**
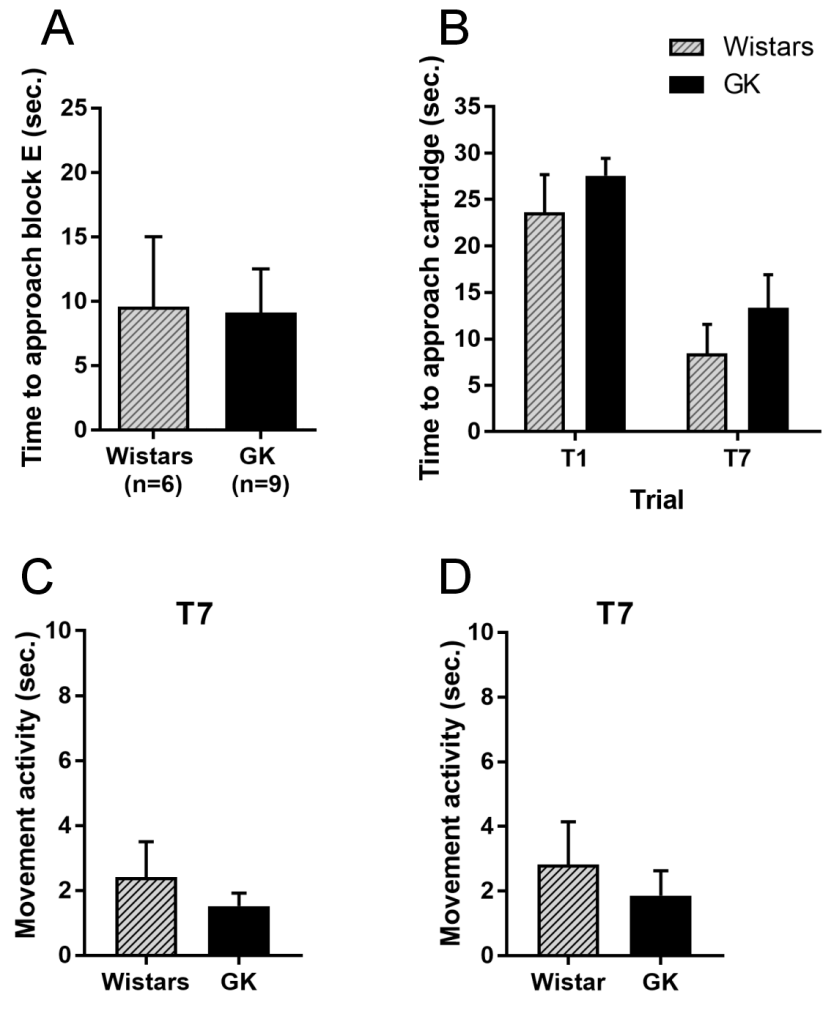
**

**Fig. S3 Diabetes does not influence the time to approach the fragrant object and the movement activity in the block test (A, C) and the habituation-dishabituation test (B, D).** The data, presented from trial (T) 7 (A, C, D) as well as T1 and T7 (B), are means ±S.E.M., (n=9-11)

**
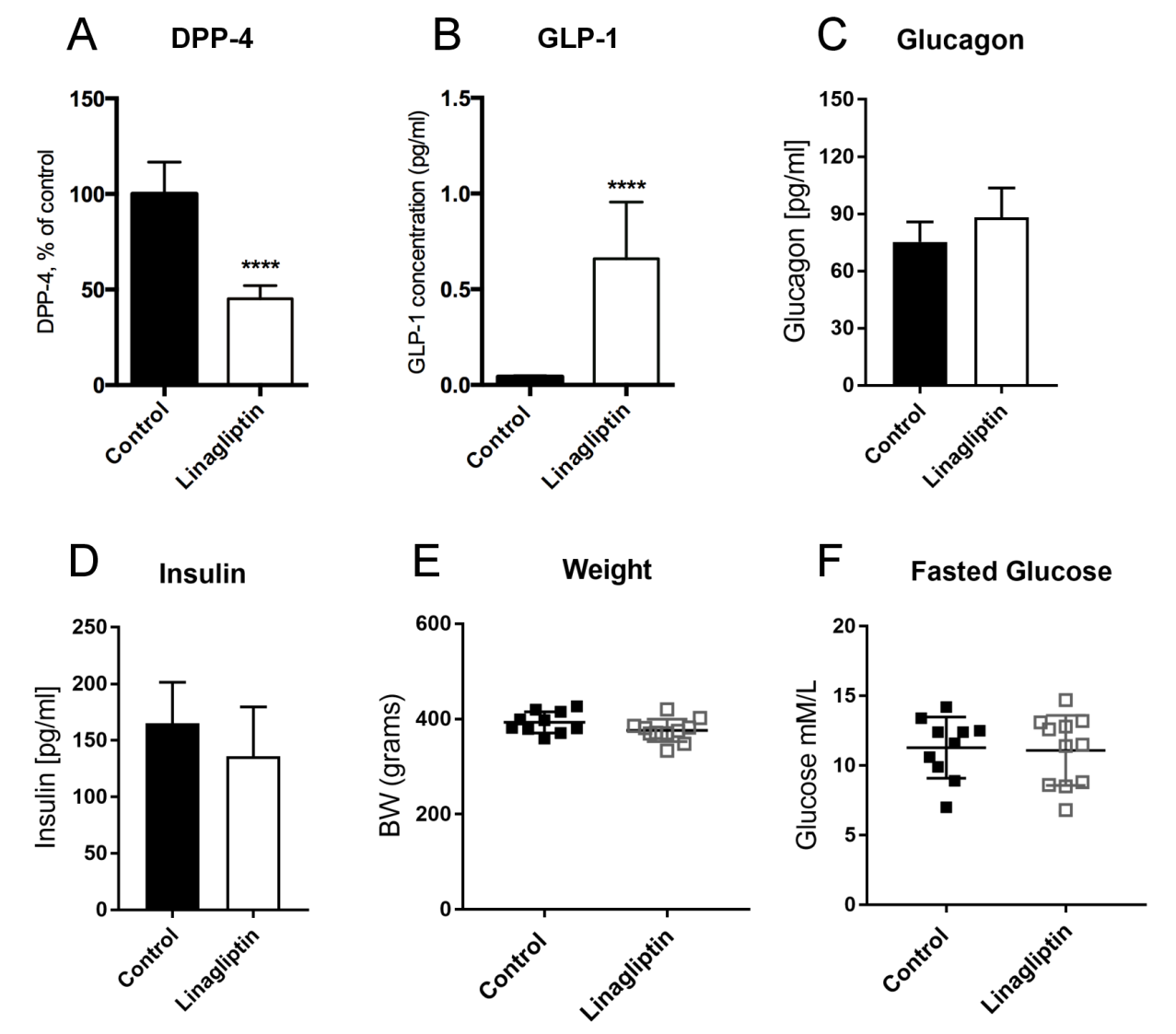
**

**Fig. S4 Dipeptidyl peptidase-4 (DPP-4) activity (A), glucagon-like peptide-1 (GLP-1) concentration (B), and metabolic parameters: glucagon (C) and insulin (D) concentration, body weight (E) and glucose concentration (F) after 8 weeks of DPP4 inhibition (Study2).** Control GK (n=10) vs. linagliptin-treated GK (n=11) rats. Two-tailed, unpaired *t*-test. The data are means ±S.E.M., **** p <0.0001
